# BioGraph: a web application and a graph database for querying and analyzing bioinformatics resources

**DOI:** 10.1186/s12918-018-0616-4

**Published:** 2018-11-20

**Authors:** Antonio Messina, Antonino Fiannaca, Laura La Paglia, Massimo La Rosa, Alfonso Urso

**Affiliations:** CNR-ICAR, National Research Council of Italy, Via Ugo La Malfa, 153, Palermo, Italy

**Keywords:** Integrated databases, Bioinformatics databases, Graph databases, BioGraphDB, miRNA

## Abstract

**Background:**

Several online databases provide a large amount of biomedical data of different biological entities. These resources are typically stored in systems implementing their own data model, user interface and query language. On the other hand, in many bioinformatics scenarios there is often the need to use more than one resource. The availability of a single bioinformatics platform that integrates many biological resources and services is, for those reasons a fundamental issue.

**Description:**

Here, we present BioGraph, a web application that allows to query, visualize and analyze biological data belonging to several online available sources. BioGraph is built upon our previously developed graph database called BioGraphDB, that integrates and stores heterogeneous biological resources and make them available by means of a common structure and a unique query language. BioGraph implements state-of-the-art technologies and provides pre-compiled bioinformatics scenarios, as well as the possibility to perform custom queries and obtaining an interactive and dynamic visualization of results.

**Conclusion:**

We present a case study about functional analysis of microRNA in breast cancer in order to demonstrate the functionalities of the system. BioGraph is freely available at http://biograph.pa.icar.cnr.it. Source files are available on GitHub at https://github.com/IcarPA-TBlab/BioGraph

## Background

The introduction of high-throughput technologies, together with bioinformatics support, has revolutionized the biomedical field paving the way for integrated approaches aimed to solve biomedical tasks. In the last years, challenges in bioinformatics have increasingly become more complex, experiencing a transition from single-task to multi-task and multi-level problems. The knowledge extracted from biological and medical data has been collected and stored in publicly available databases, to give scientists the possibility to analyze and visualize data through information systems. Hundreds of different databases are freely available to the scientific community for the analysis of a large amount of biomedical data. These systems collect both experimentally validated and computationally predicted data, as well as attributes and relationships among biological entities. Although many advances have been made during this last decade, there are still difficulties in exploring and analyzing data derived from multiple resources. These problems are due to different factors, such as the use of various platforms and frameworks, the coexistence of heterogeneous query languages and data formats, the lack of a standard data storage and nomenclature, and, finally, the presence of multiple resources for the same kind of data. For instance, a typical bioinformatics scenario in the translational medical field is the functional analysis of microRNA (miRNA) molecules in cancer pathology; microRNA are small non-coding RNA molecules with a regulative role in gene expression [1]. They have been investigated in cancer as potential biomarkers and targeted therapy for cancer treatments [2]. For the functional analysis of miRNA, different databases have to be used, each of them with a different interface, structure, storage system, and query language. Consequently, the output produced is a complex holder of the different information, because each of them requires a different approach to be handled properly. To overcome the drawbacks of a handcrafted combination of different resources, some efforts have been made in developing services and databases that integrate biomedical and biological publicly available resources.

An example of an open-source framework that allows to import and integrate different public biological data sources into a data warehouse is Java BioWareHouse (JBioWH) [3]. This SQL-based framework defines a set of data types related to bio-entities, such as genes, proteins, pathways, and drugs. JBioWH allows the unskilled users to use some graphical queries through the desktop client tool. Meanwhile, it is possible to write and execute simple SQL queries with the command line tool. To perform all the complex queries that can not be easily defined in SQL language, it offers a powerful Java library.

InterMine project [4] is another interesting platform developed with the aim of integrating and analyzing heterogeneous biological data. It defines an open-source data warehouse system and a powerful engine for building custom bioinformatics queries. Several public web-services have been developed using the InterMine project, such as FlyMine [5], MedicMine [6] and HumanMine [7]. In particular, the last one integrates some *Homo Sapiens* genomic data, including genes, proteins, miRNA, pathways, diseases, and functional associations.

Bio4J [8] integrates biological data exploiting a distributed graph database, where each biological entity is represented as a node and the relationship (and their attributes) among two entities is represented as an edge. Bio4J defines a framework where connections among all the largest publicly available repositories in the field of proteins, genes, enzymes and biological pathways of the human species, are integrated. Bio4J users can perform different kinds of search because it supports query languages allowing both declarative and traversal queries.

Also, some particular problems require performing a precise analysis from various publicly available databases. For instance, miRWalk 2.0 [9] integrates biological resources exploiting a relational database. It collects predicted and manually validated miRNA-target interactions, and their related biological entities and processes in human, mouse and rat species. miRWalk defines some pre-defined search methods, which are used for querying its database; it provides annotations and mine relationships among integrated data, such as miRNAs, genes, diseases, and pathways.

The non-coding RNA human interaction Data Base (ncRNA-DB) [10] is another integrated database that aims at collecting data for a specific problem, i.e. the reconstruction and the visualization of non-coding regulatory networks. As well as miRWalk, it collects genes, pathways, and disease data from public on-line repositories, but it also integrates ncRNAs data interactions from a large number of availablerepositories.

An example of a more specific purpose integrated database is the Adipogenic Regulation Network (ARN) [11] that allows performing the analysis and the prediction of the adipogenesis process. More in detail, it integrates genes, miRNAs and their adipogenic regulation implications from genomics and literature public databases. ARN also provide a web-service that permits to generate and evaluate hypotheses for putative target control approaches. Data is stored in a flexible and performing NoSQL database that also provides a Java API for querying the on-line ncRNA-DB web service, whereas it can also be used as a server for third party client applications.

Another class of problems requires analyzing only a specific biological information, such as the functional gene annotation or the species biochemical reactions. Often, since the same kind of information is contained in more than one on-line resource, it is necessary to gather and standardize data from different available resources. An example of this kind of integrated database is the species specific essential reactions database (SSER) [12]. It gives users a centralized repository and a web service that allows to search, compare, and download all the collected biochemical reactions of twenty-six organisms, to explore metabolic network models and discover drug targets.

In this work we present BioGraph, a new web app for querying and analyzing biological entities. BioGraph is built upon our previous published graph database called BioGraphDB [13–15], which collects and integrates heterogeneous biological data. In particular, BioGraph allows to perform queries using a single query language and format about all the biological resources stored into BioGraphDB; it provides a web-user interface, an interactive and dynamic visualization of the results; the pre-defined implementation of queries related to common bioinformatics scenarios; the possibility to create custom queries; the possibility to export the results in the most common data formats.

Further in this paper, we will analyze the main features of BioGraph web app and then we will compare it with some of aforementioned integrated databases.

## Construction and content

In this section, we present the technical features and the information content of the proposed system. First of all we introduce the biological entities and the data sources we considered; then we describe the software modules used to download and integrate all the data. The last four subsections are, respectively, about the structure of the underlying graph database, the description of the adopted query language, the architecture of the proposed web application and the web user interface and its features.

### Data sources

At present, BioGraphDB is composed of the following biological and bioinformatics resources.

- Entrez Gene
  NCBI Entrez Gene database [16] represents one of the richest collection of information related to genes belonging to fully sequenced genomes. Entrez Gene has information about gene products and their properties, nomenclature, gene location, phenotypes, sequences, set of homologs and orthologs, variation details, expression.
- UniprotKB
  Uniprot Knowledge Base (UniprotKB) [17] is the largest freely accessible bioinformatics database about protein sequences and their annotations. It is considered the main hub for proteomic data, including information such as protein name and its description, amino acid sequence, taxonomic data, and accurate annotations.
- miRBase
  microRNA database (miRBase) [18] is the complete repository of sequences and annotations of microRNA (miRNA). It includes both the precursor and mature sequences of more than 200 species.
- HGNC
  HUGO Gene Nomenclature Committee (HGNC) [19] is the institution that established the gene nomenclature for the human species. HGNC database, therefore, provides for each gene its official name, also known as gene symbol, as well as a list of corresponding identifiers in other genomic databases, such as RefSeq and Entrez Gene for instance. This way, HGNC is the best source for disambiguation of a gene, and protein, names and identifiers.
- GO
  Gene Ontology (GO) [20] is the most popular framework describing gene functions regarding molecular function, cellular component and biological process. GO defines concepts and annotations.
- Reactome
  Reactome [21, 22] is, along with Kegg [23], the reference database for the collection and annotation of molecular pathways. It stores validated pathway related to the human species and computationally predicted pathways for about 20 other species. Reactome has been selected rather than Kegg because the former is freely downloadable.
- miRCancer
  miRCancer database [24] is an open access repository of associations between deregulated miRNAs and human cancer extracted from Pubmed literature. An association is first discovered by using text mining techniques and then it is manually confirmed.
- miRNASNP
  miRNASNP [25] is a database that stores information about the effects of single nucleotide polymorphism (SNP) in miRNA-target interactions.
- miRNA-Target Interaction
  This last one is actually a collection of both manually verified and predicted interactions between miRNAs and their mRNA target. In particular it was considered miRTarBase [26] for the verified interactions, and mirWalk [9] and miRanda [27] for the predicted interactions.

### Extract, transform, and load tools

All the earlier cited data sources are available for download. To somehow automate the download and the data import processes to build an updated brand-new instance of BioGraphDB, some tools have been developed.

The download process is supervised by a shell script, which uses a set of standard Unix command line utilities to perform some basic operations, such as transfer, decompression, filtering, and extraction of relevant biological data.

Many of the obtained data files are supplied in textual tab-separated values format, where each line of the text file is a record, and each field value of a record is separated from the next by a tab character. By contrast, miRBase, GO, and UniprotKB are available in EMBL text file format [28] and XML format.

To efficiently manage the complexity and the extreme abundance of available data and external references, a modular Extract-Transform-Load (ETL) tool processes source data. A precise order of execution of ETLs sub-modules guarantees data consistency and proper relations between entities. This way, when a data source which refers to others is imported, the database already contains all the depending resources.

### Database schema

Graph data modeling is the process in which an arbitrary domain is described as a connected graph of nodes and relationships.

**Fig. 1:**
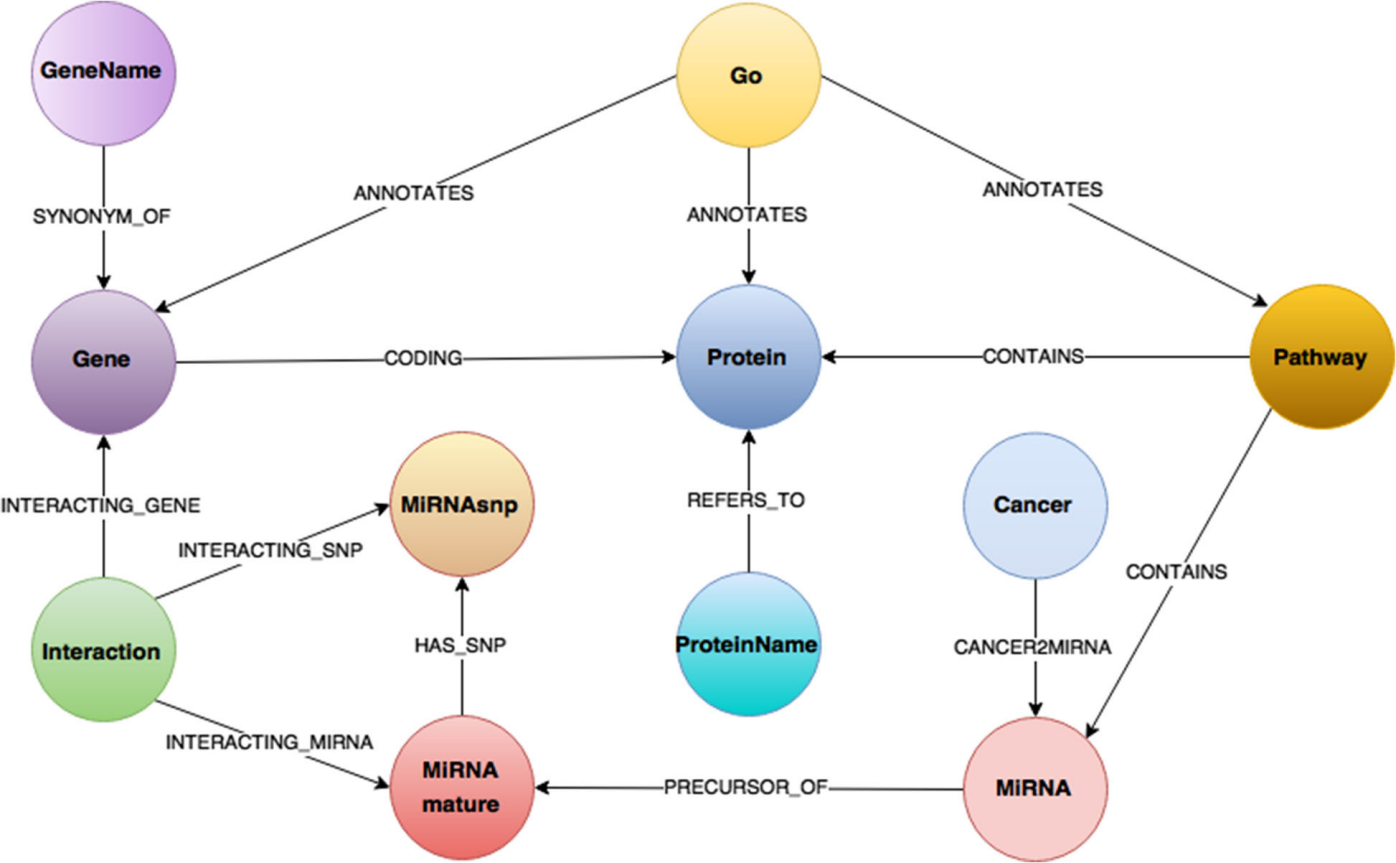
BioGraphDB scheme. The graph data model of BioGraphDB consists of a set of nodes/vertices classes matching the biological entities provided by the used data sources. Relationships between entities are modeled as relations, or edges

In our data sources, almost all entities and references are already well identified. Therefore, it is quite easy to give an abstract representation of BioGraphDB database [13], as shown in Fig. 1. A simple general rule has been followed: any biological entity has been mapped into a node with attributes, and a relationship between two biological entities has been mapped into a relation. According to the nature of the entities, nodes and relations have been grouped into classes, each identified by a label. For example, all the genes imported from Entrez Gene become nodes identified by the label *Gene* and all the proteins read from the Uniprot Knowledge Base become nodes identified by the label *Protein*. At this point, the relation *CODING* between genes and proteins can be created using the information on this relationship from HGNC.

Table 1 summarizes all associations between the biological information and the created graph entities. At present, BioGraphDB contains about 1,450,000 nodes and 2,820,000 relations.

**Table 1:** Associations between graph entities and biological information

*Type*	*Label*	#	*Biological information*	*Source*
Nodes	Gene	59839	Genes	NCBI entrez genes
	Go	43969	Functional annotations	Gene ontology
	Protein	20193	Proteins	UniProtKB
	Pathway	1920	Pathways	Reactome
	MiRNA	28645	miRNA precursors	miRBase
	MiRNAmature	38558	miRNA matures	miRBase
	MiRNAsnp	236	miRNA SNPs	miRNASNP
	Cancer	107	Cancers	mirCancer
	ProteinName	219132	Proteins accessions	UniProtKB
	GeneName	115027	Genes symbols	HGNC
	Interaction	913285	miRNA-target interactions	mirTarBase,miRanda
Relations	ANNOTATES	514528	Links to annotated entities	Gene ontology
	CONTAINS	99979	Links to entities in pathways	Reactome
	PRECURSOR_OF	38558	Precursors-matures relations	miRBase
	HAS_SNP	236	miRNAs-mutations relations	miRNASNP
	SYNONYM_OF	115027	Symbols-genes relations	HGNC
	REFERS_TO	219132	Accessions-proteins relations	UniProtKB
	INTERACTING_GENE	913285	Genes-interactions relations	mirTarBase,miRanda,miRNASNP
	INTERACTING_MIRNA	657904	miRNA-interactions relations	mirTarBase,miRanda
	INTERACTING_SNP	255381	SNPs-interactions relations	miRNASNP

### Gremlin query language

Gremlin [29] is the graph traversal language of Apache Tinkerpop [30], a very popular open-source and vendor-agnostic graph computingframework. At present, Apache Tinkerpop is supported by the most important graph systems available in the market. Language drivers are also available for almost all languages. Gremlin is a functional, data-flow language designed according to the “write once, run anywhere”-philosophy, to analyze and manipulate property graphs, which are graph data structures characterized by the following

- both vertices and edges can have any number of properties associated with them;
- edges in the graph have a directionality;
- there can be many types of edges, and thus, many types of relationships can exist between the vertices.

Every Gremlin traversal is composed of a sequence of steps, able to perform atomic operations on the data stream. Those steps can be *transform-based* (they take an object and emit a transformation of it), *filter-based* (to decide whether to allow an object to pass or not), *sideEffect-based* (they pass the object, but yield some side effect), and *branch-based* (to decide which following step to take).

A Gremlin traversal can be written in a declarative, in an imperative, or in a mixed manner containing both declarative and imperative aspects. With the declarative way, you do not tell the traverses the order in which to execute their walk: a traverse is allowed to select a pattern to execute from a collection of other patterns. Instead, an imperative Gremlin traversal tells the traverses how to proceed at each step in the traversal.

If applied to big integrated bioinformatics graph databases, imperative traversals are suitable to easily and properly state the common or user-defined bioinformatics tasks that a biologist has to address in his daily work. It means that many typical bioinformatics scenarios can be solved by a more or less complex Gremlin query. Every scenario can usually be decomposed in a row of simple sub-tasks, easily translatable into a few Gremlin steps. Definitively, a scenario can be meant as a graph traversal operation, and Gremlin is an ultimate tool to perform such task.

It is important to emphasize that any Tinkerpop-enabled graph systems can execute Gremlin traversals. Furthermore, not only every Gremlin traversal is suitable for online transactional processing (OLTP) as a real-time database query, but it is also useful for online analytical processing (OLAP) as a batch analytics query.

### Web application architecture

**Fig. 2:**
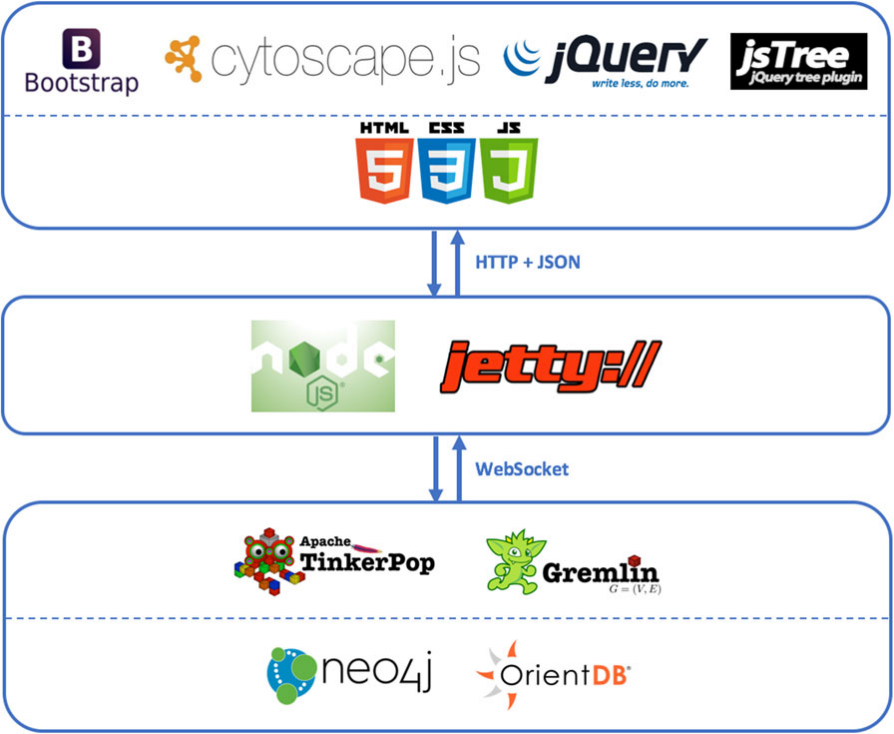
BioGraph architectural stack. The pictures gives an overview on the state-of-the-art technology behind BioGraph. Used tools are grouped into three levels. From the bottom to the top: the Graph Data level overlooked by Apache Tinkerpop, the Microservices level, and the Web Application level. Protocols and data formats of inter-level communications are also highlighted

The application’s architecture is highly modular and scalable and makes use of many state-of-art technologies to ensure responsiveness and performances. The application has the full stack architecture shown in Fig. 2.

The web user interface is mainly built with Bootstrap [31], a popular HTML, CSS, and JavaScript framework originally developed by Twitter to easily and quickly create responsive web front-ends. By contrast, dynamic content manipulation, event handling, effects, animations, and asynchronous data transfers are managed by jQuery [32].

Graphs visualizations and interactions are handled by Cytoscape.js [33], a powerful JavaScript library which allows easy display and manipulation of rich interactive graphs. It supports both desktop and mobile browsers, and it natively includes all the common gestures, such as panning, box selection, pic-to-zoom, etcetera.

The middleware of the application consists of a set of microservices based on Node.js [34] and Jetty [35]. Some deal with the management, transformation, and production of queries results emitted by the graph engine. Some others are needed to implement the word auto-completion features and the production of *p*-values calculated using the right-tailed Fisher exact test [36].

The bottom of the stack is composed of the graph computing framework Apache Tinkerpop 3 [30] with its Gremlin Server, which provides a way to remotely execute Gremlin queries against graph instances hosted within it. At present, BioGraphDB is built as a Neo4j [37] instance. An OrientDB [38] instance is under development and it will be released when OrientDB 3 will be officially available. Latest available release of OrientDB, in fact, still does not support Apache Tinkerpop 3.

### Web user interface

**Fig. 3:**
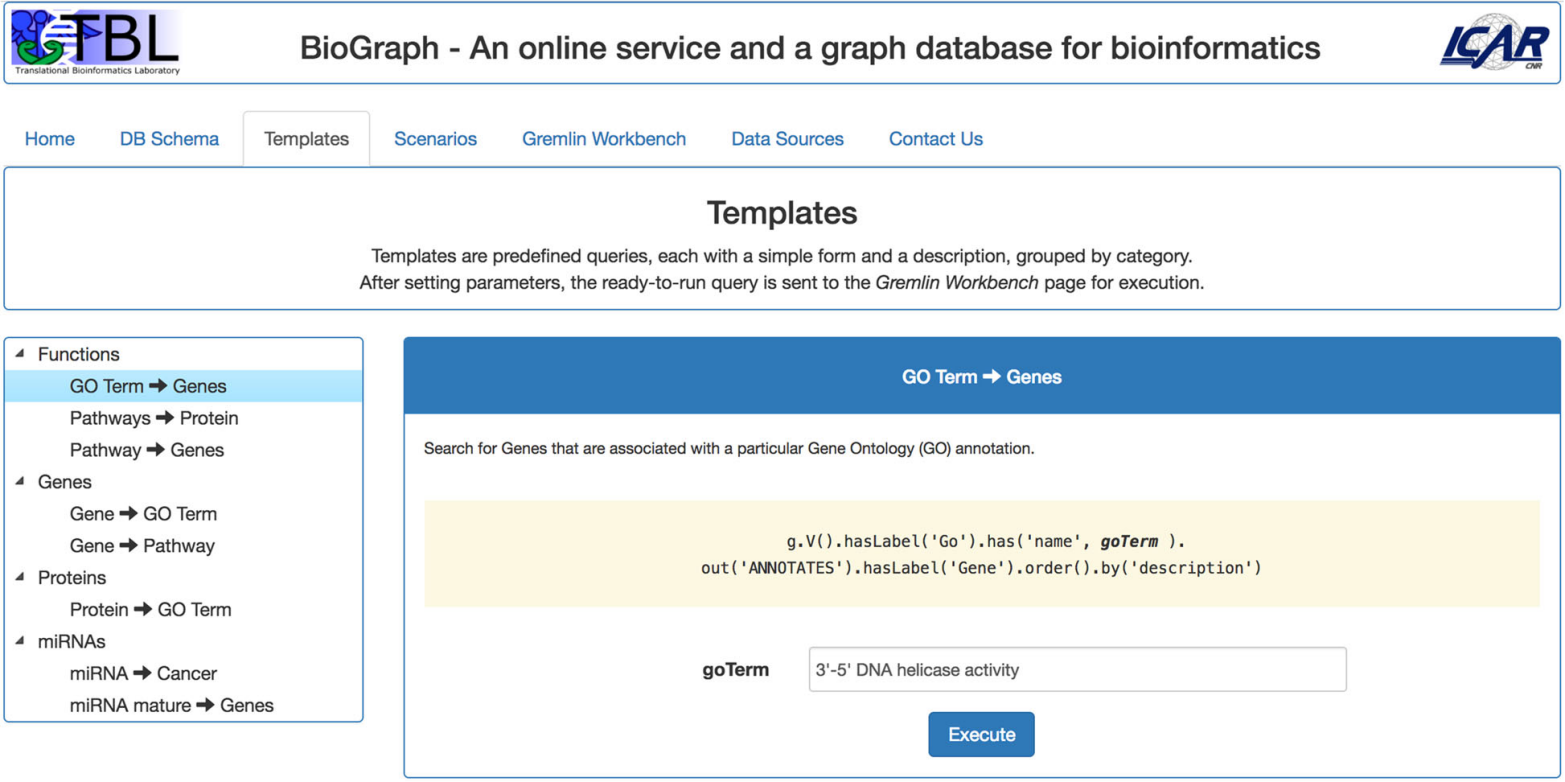
The Templates tab. Templates are simple predefined queries given as examples of how an user can traverse BioGraphDB. The queries are customizable and grouped by category
- *Scenarios* contains, at present, four predefined complex queries, proposed as example of how BioGraph and Gremlin can help in the analysis of specific non-trivial problems. The available scenarios, as shown in Fig. 4, are:

Web User Interface (WUI) is organized in the following set of tabs:

- *Home* contains the website’s welcome landing page.
- *DB Schema* presents the BioGraphDB graph model in Fig. 1, plus detailed information on all properties of nodes and relationships.
- *Templates* proposes a set of simple predefined queries, grouped by the following categories: Functions, Genes, Proteins, and miRNAs (see Fig. 3). Each template accepts one or more parameters and the *Execute* button sends the related query to the Gremlin Workbench for execution.

**Fig. 4:**
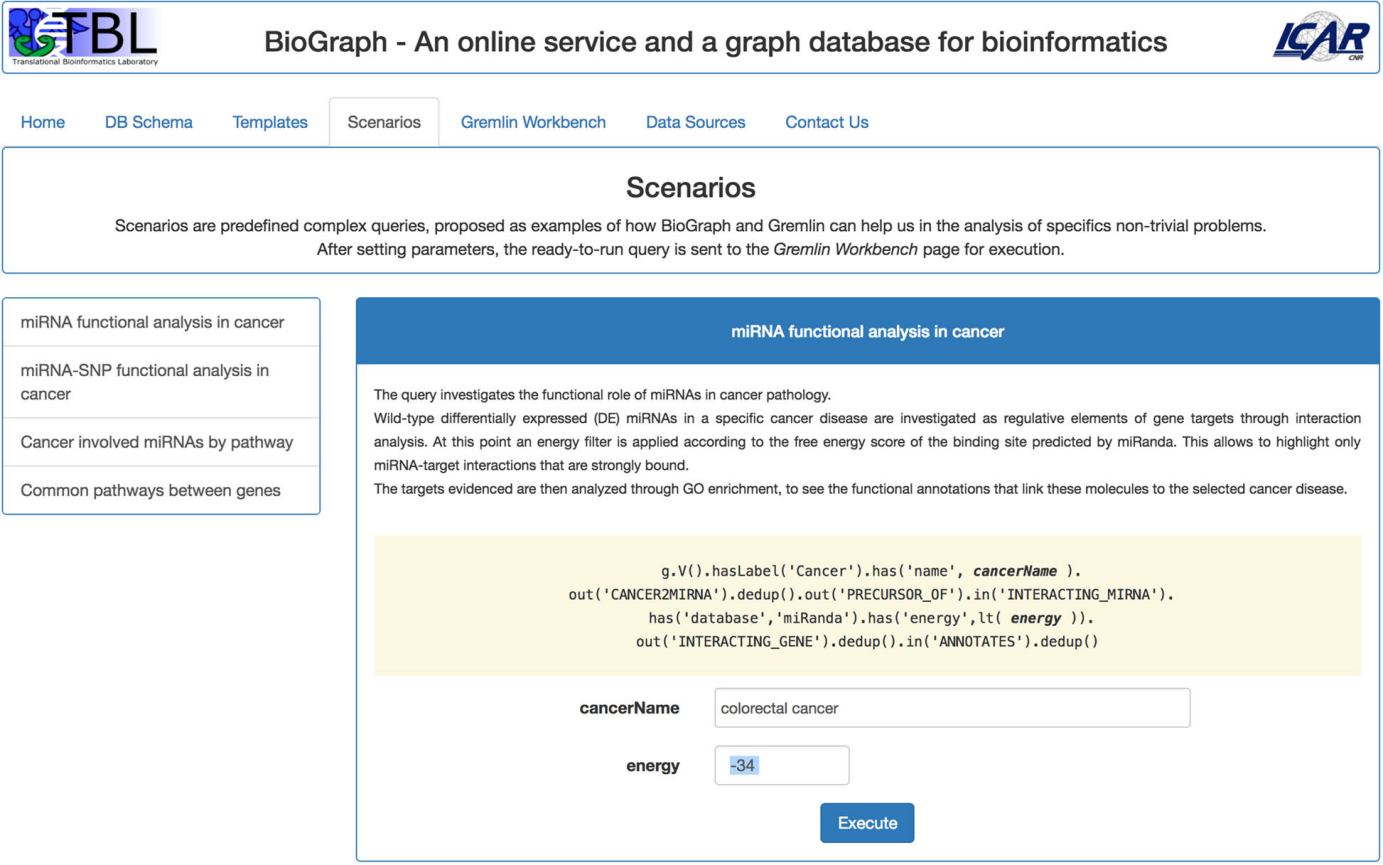
The Scenarios tab. The four proposed scenarios are examples of how complex Gremlin queries can help in the analysis of non-trivial bioinformatics problems

**Fig. 5:**
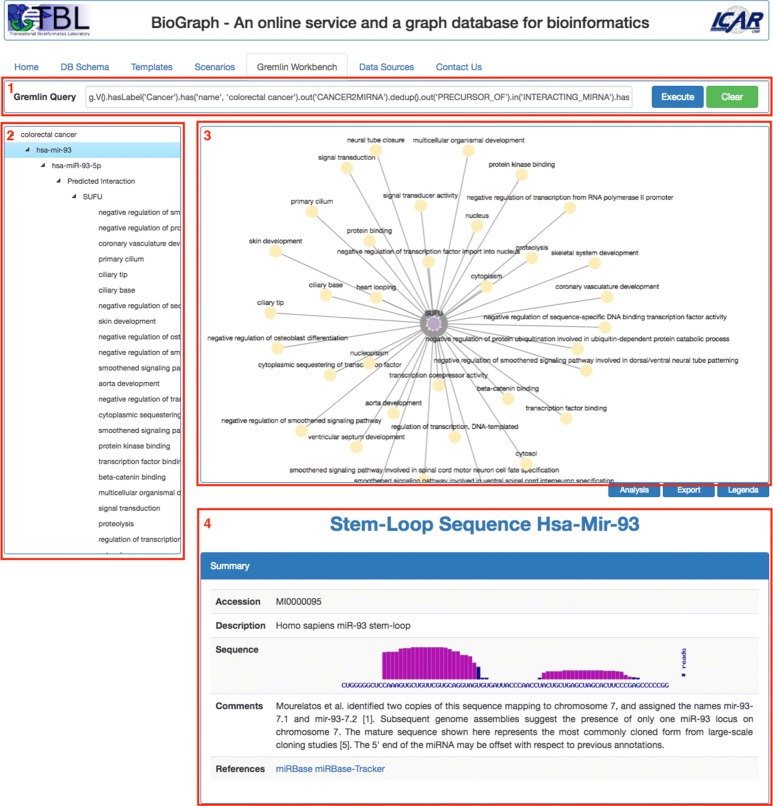
The Gremlin Workbench. It is basically composed of four panes: 1) the Gremlin query pane, 2) Tree View, 3) Graph view-port, 4) the Details pane

  - miRNA functional analysis in cancer;
  - miRNA-SNP functional analysis in cancer;
  - Cancer involved miRNAs by pathway;
  - Common pathways between two genes.
  Again, some parameters can be set before the execution and queries results are automatically shown in the Gremlin Workbench tab.
- *Gremlin Workbench* is the place where most of user’s activities are performed. It is shown in Fig. 5 and consists of the following main panes:

  - *Gremlin Query*, where the user can type and send for execution a Gremlin traversal query over BioGraphDB;
  - *Tree View* contains an interactive tree that shows all the crossed nodes and edges produced by the Gremlin traversal query. The tree is built with the jQuery plugin jsTree [39], which uses jQuery’s event system and supports HTML/JSON data sources and AJAX loading;
  - *Graph View-port* displays queries results as interactive graphs. Several gestures are supported, such as pich to zoom, mouse wheel to zoom, tap to select, tap background to unselect, grab and drag of nodes. Selecting a node triggers the immediate visualization of all related node information in the Details pane;
  - *Details* provides detailed information on a selected item. The pane’s layout and contents strictly depend on the type of the item. For example, for a cancer, it presents a summary extracted in real-time from the related Wikipedia page, followed by the list of all linked miRNAs formatted as a browsable table. Or again, for a miRNA mature, the summary contains the accession and the sequence, while the targets are grouped in two browsable table below, with validated target first, followed by predicted targets.
  Other features are also available via buttons under the Graph View-port:
  - *Analysis* lets the user calculate the *p*-values when the results contain pathways and proteins (or genes), functional annotations and proteins, or functional annotations and genes.
    The *p*-values are calculated using the right-tailed Fisher Exact Test.
  - *Export* lets the user export the graph results in the following formats: TSV (the simple tab-separated values text file format), GraphML (an XML-based file format for graphs that supports many graph structures, including the directed property graph structure used in this work), JSON (useful to import the results into Cytoscape [40]), and PNG (to obtain an image based on the current content of the graph viewport).
  - *Legenda* simply displays a key of colors of the biological entities in the results graph.
- *Data Sources* shows information about the imported integrated databases, such as name, version, and source URL.
- *Contact Us* activates a simple form to contact the authors and it is useful to receive users feedbacks on the use of the service.

## Utility and discussion

In this section we describe the main functionalities of BioGraph. First of all we propose a case study in order to show how the system can be used to solve a bioinformatics scenario. Moreover we compare BioGraph with other similar integrated databases and services already introduced in “Background” section. The comparison is carried out considering both technical and functional aspects. Finally we provide all the information about the software availability.

### Case study: functional analysis of microRNAs in breast cancer pathology

The last decade has increasingly seen the emerging role of microRNAs (miRNAs) as biomarkers in different diseases, and cancer hallmarks like adhesion, proliferation, translation and inflammation [41]. In particular, since some specific cancer subtypes or cancer hallmarks are strictly related to miRNAs, the use of these miRNAs could be taken into account for future targeted therapies. Moreover, breast cancer (BC) studies proved the involvement of miRNAs in tumour progression and metastasis [42], as they result in differentially expressed (DE) tumour samples compared with healthy tissues [42, 43]. However, functional analysis of these small RNA samples needs to be deeply investigated, to validate their actions as diagnostic biomarkers in this disease. To this aim, research has been focused on putative miRNA targets, and on gene enrichment analysis. Many tools and algorithms, based on different features, have been developed to further this aim [44].

In this case study, we exploit the proposed BioGraph web application to investigate the role of DE miRNAs in breast tumour samples through Gene Ontology (GO) [20] analysis. This study aims to give a functional significance to, and consequently to investigate the potential role of, those DE miRNAs that are related to some clinical features of BC. To solve the functional analysis of microRNAs in breast cancer pathology, many tools and online services are needed: after choosing cancer pathology, DE miRNAs have to be selected through a repository as miRCancer [24]. After that, differentially expressed miRNAs will be used to evidence miRNA-target interaction, through dedicated databases as miRanda [27]. At this point, a list of targets is obtained as result. Finally, to evidence the functional annotations linked to these targets, the GO database is needed. Each step of this analysis requires, as previously said, a different database, with different features, interfaces, storage system. Moreover, every intermediate result must be saved, converted, and loaded again somewhere.

**Fig. 6:**
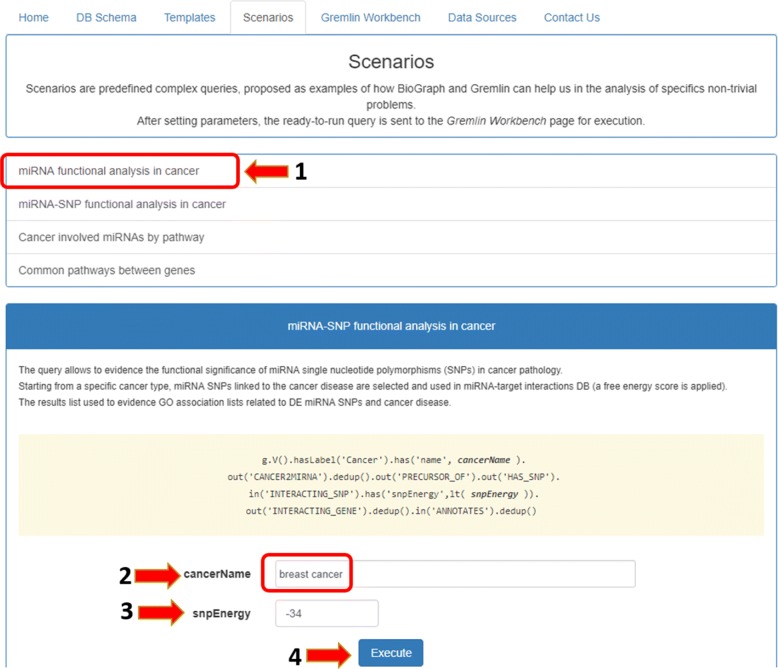
Case study scenario. The functional analysis of miRNAs in breast cancer can be done starting from the first scenario in the Scenarios tab and personalizing the values of parameters
- manually typing a proper gremlin query (Fig. 7).

BioGraph allows to avoid all these annoying processes, simply choosing the starting point and indicating the sequence of resources to use. We briefly describe all the steps needed to solve the proposed scenario. First of all, it is necessary to define a query; it can be done in two ways:

- using and customizing one of some predefined complex queries (Fig. 6);

**Fig. 7:**
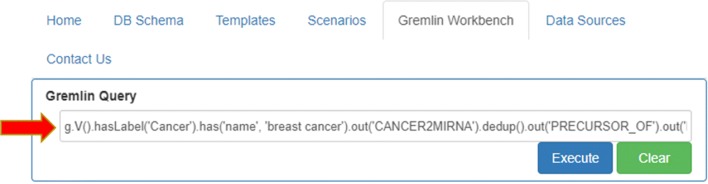
Custom Gremlin query. Gremlin Workbench allows the user to manually type and execute any query he wants

**Fig. 8:**
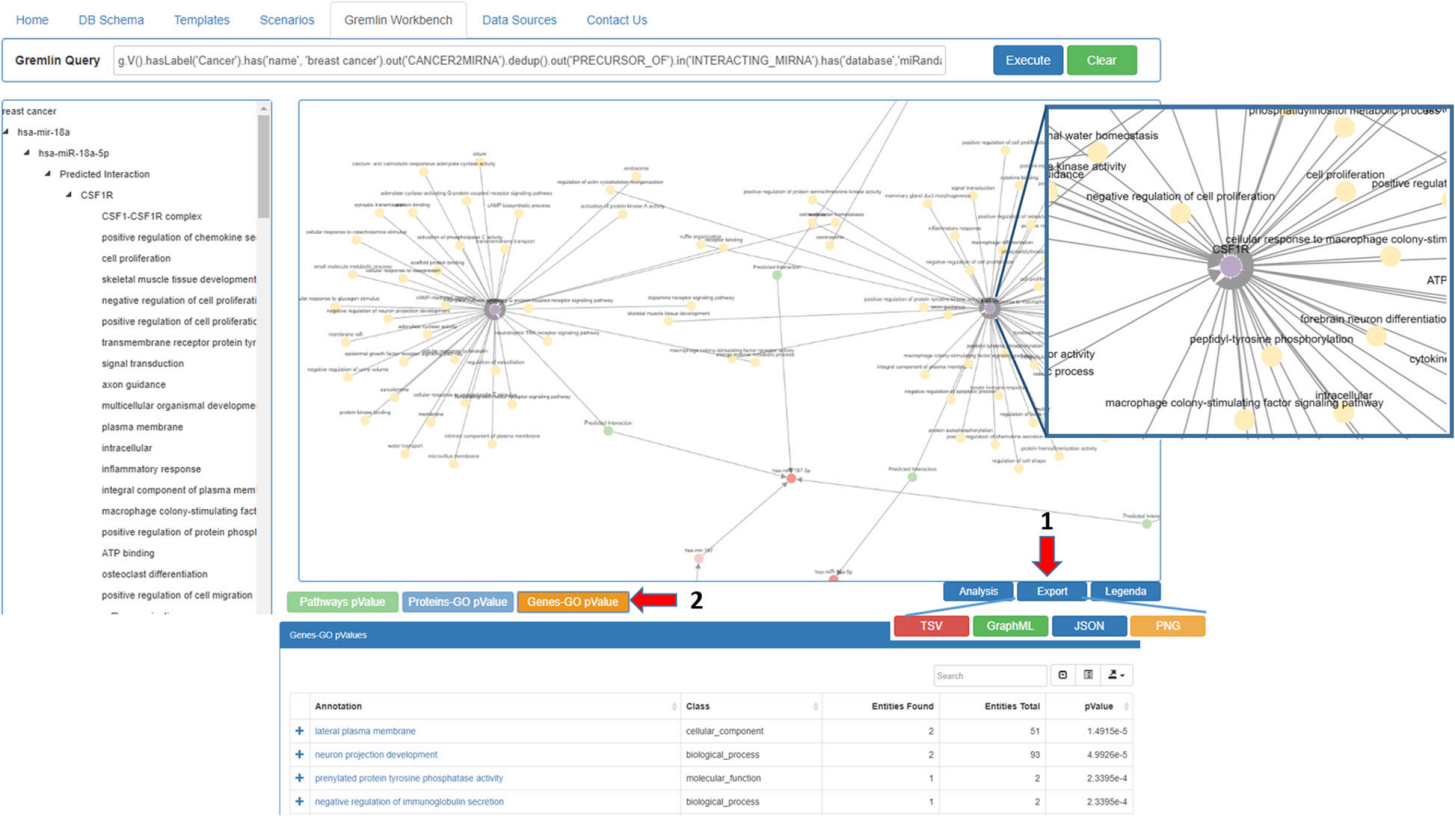
Results for the case study scenario. The user can immediately study the results given in graphical and tree form. He can also (1) export them in several formats or (2) perform some data analysis, when applicable

**Fig. 9:**
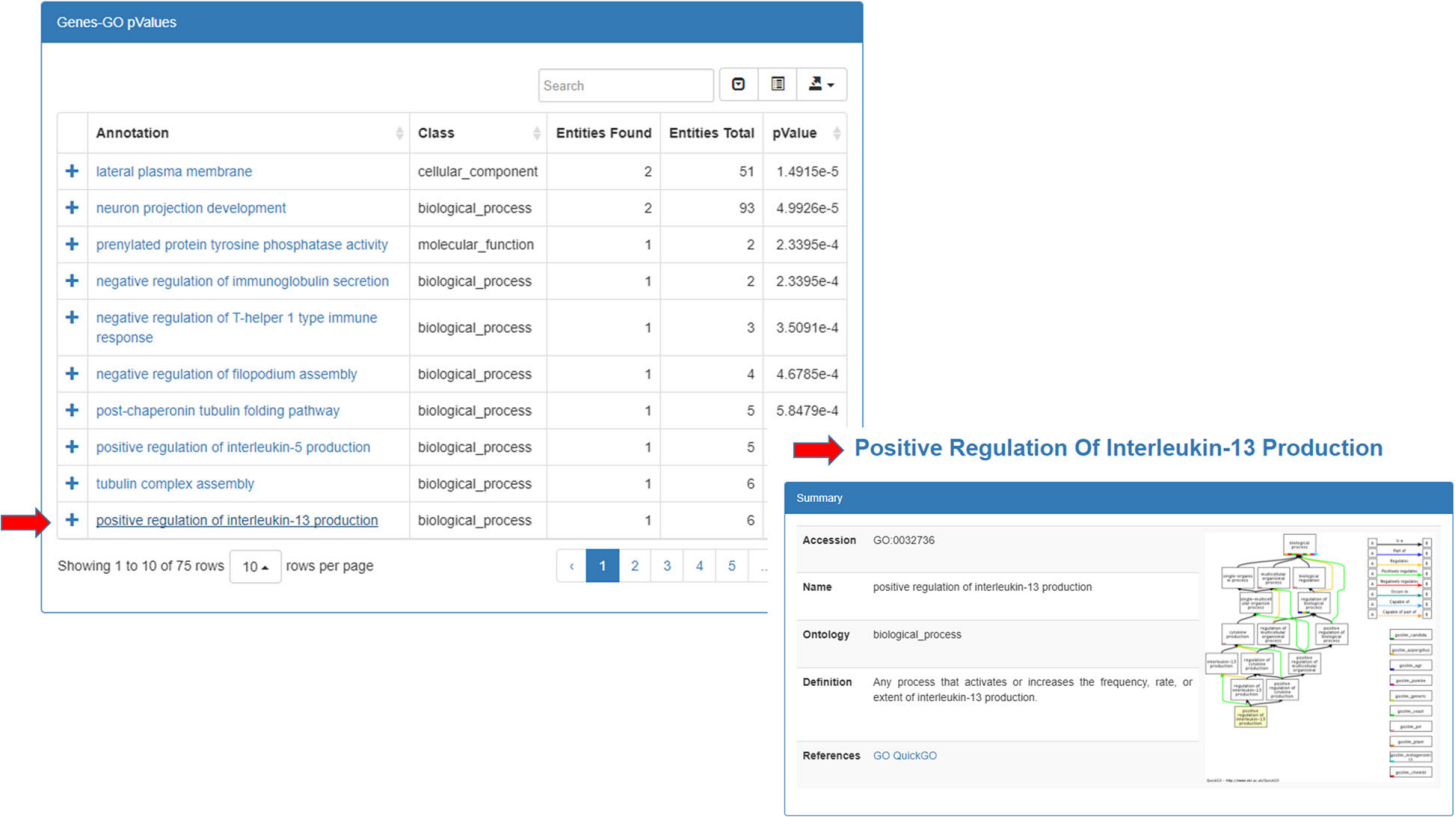
GO analysis Analysis of data through *p*-value calculation. As the figure shows, it is possible to deeply investigate a specific field of the interrogation. In the shown case study figure, it is possible to investigate about a specific annotation linked to a particular gene target

In the first case, users can activate the *Scenarios* tab, select the “miRNA functional analysis in cancer” case study on the left, and then set the values of the fields “cancer name” and “energy” filter. In the second case, users can directly activate the *Gremlin Workbench* to manually write a custom Gremlin query. In both cases, to run queries, the *Execute* button must be pressed. As result, the system will report the complete answer automatically both in graphic and in tree form. The interactive visualization allows showing details about each node of the graphical result, as well as to navigate through resulting path. Since the query results (leaves of the graph) are miRNA-target functional annotations, the *Analysis* function enables users to press the “Gene-GO *p*-value” button, as shown in Fig. 8. If users want to visualize a detail of annotation field, they just have to select the term of interest, and the information related to the selected GO term will be displayed (Fig. 9). Results can also be exported for further processing.

### Comparison with related web applications and databases

In order to compare our proposed BioGraph system with other integrated databases in bioinformatics domain, we took into account two different perspectives. The former considers the technological point of view, highlighting the type of DB and DBMS adopted, the availability of source codes, ETL or API and the kind of query language. The latter is related to contents and services provided by DBs, including the data types, the presence of a web interface, the possibility to make custom queries, the availability of dynamic visualization and analytics functionalities, the possibility to expand the system with new data sources or services. The comparison has been done with the integrated DBs already introduced in “Background” section, namely ncRNA-DB, JBioWH, mirWalk, ARN, SSER, Bio4j, and HumanMine.

The features described above have been summarized in Table 2 (technical perspective) and Table 3 (contents and services perspective). Starting from Table 2, it is possible to note that only 3 out of 8 systems, BioGraph, Bio4j, ncRNA-DB, are based on NoSQL graph databases. Those three systems, although sharing the architecture of the database, adopt different query languages. In particular ncRNA-DB uses the SQL-like queries and the Java APIs provided by the Orient platform; Bio4j implements the Anguillos query language [8], that is a custom query language developed by Bio4j developers; BioGraph uses Gremlin language, that, as explained in the previous section, allowed us to develop a system that is independent from the architecture and type of the underlying graph database. The remaining DBs, implementing a relational or object oriented database, use SQL language, apart from JBioWH that further provides dedicated APIs in order to make queries that can not be done using only SQL language. Finally only 5 out 8 databases make available their source code, the ETL or API in order to implement customized version of their products or to integrate other resources. In particular, ncRNA-dB, Bio4J and JBioWH provides Java API, HumanMine offers web service API and BioGraph makes available the source code, including ETL. Looking at the contents and services (Table 3), the most evident feature is the kind and number of data sources (first column). There we can see how there are databases most concerned with few or only one data type, e.g. SSER that only considers data about essential reaction; whereas there are other systems, such as our BioGraph, Bio4j, JBioWH, that integrates and make accessible within a single platform many heterogeneous biomedical and bioinformatics data. In the second column we report the presence of a web interface to access the DB, with the only exception of JBioWH, that provides a desktop client, and Bio4j that provides a programmatic access. The third column shows the presence or less of a dynamic visualization, rather than simple table visualization. By dynamic visualization, we meant that the visualized results are highly interactive and can be manipulated. For example, BioGraph gives as results a graph that can be arranged by the user and, moreover, its nodes and edges can be expanded in order to provide further information. Custom query, fourth column, means the user can personalize the input query, rather than using pre-defined forms or templates. Analytics, fifth column, means the system provides some sort of data analysis (e.g. *p*-value computation). Finally expandability, last column, specifies if the system can be updated with more recent data or if new resources can be added.

**Table 2:** Technical and technological features of BioGraph and other considered integrated systems

*Database*	*Database type*	*DBMS*	*Sources/ETLs/API*	*Query language*
ncRNA-DB	Graph	OrientDB	Yes	OrientDB OSQL
JBioWH	Relational	MySQL	Yes	SQL, JBioWH API
mirWalk	Relational	MySQL	No	SQL
ARN	Relational	MS SQL Server	No	SQL
SSER	Relational	MySQL	No	SQL
Bio4j	Graph	Titan	Yes	Anguillos
HumanMine	Object oriented	PostgreSQL	Yes	SQL
BioGraph	Graph	Neo4j	Yes	Gremlin

**Table 3:** Content type and functional features of BioGraph and other considered integrated systems

*Product*	*Biological data*	*Web interface*	*Dynamic visualization*	*Custom queries*	*Analytics*	*Expandability*
ncRNA-DB	ncRNAs, RNAs, genes, diseases	Yes	Yes	Yes	No	No
JBioWH	Genes, proteins, proteins clusters, proteins domains, chromosomes, enzymes, ppi, pathways, reactions, drugs, taxonomies, functional annotations	Desktop client	No	Yes	No	Yes
mirWalk	Genes, miRNAs, functional annotations, miRNA-target interactions	Yes	No	No	Yes	No
ARN	Genes, miRNAs, regulations of adipogenesis	Yes	Yes	No	Yes	No
SSER	Essential reactions	Yes	No	No	No	No
Bio4j	Proteins, taxonomy, functional annotations, enzymes	Command line	No	Yes	No	Yes
HumanMine	Genes, proteins, protein domains, protein localizations, pathways, genes expressions, functional annotations, diseases, phenotypes, molecular interactions, genetic interactions	Yes	No	Yes	Yes	Yes
BioGraph	Genes, proteins, miRNAs, pathways, functional annotations, miRNA-target interactions, miRNA-cancer relations, miRNA-SNP relations	Yes	Yes	Yes	Yes	Yes

Considering the features summarized in Tables 2 and 3, we can say that BioGraph is a system that is up-to-date with regards to the technological solutions implemented, e.g. graph as database architecture and Gremlin as query language. Moreover, in comparison with other similar systems, BioGraph offers several services such as a dynamic visualization, the possibility to make personalized queries and a support for integrating (or updating) new biological resources.

### Software availability

BioGraph web application is available at http://biograph.pa.icar.cnr.it.

All the software needed to deploy an instance of BioGraph is released under the Apache License 2.0. The source files are available on GitHub at the URL https://github.com/IcarPA-TBlab/BioGraph and are organized as follow:

- *biograph-download* contains an example of a script to batch download all the required data sources. It decompresses original files, extracts only the useful data, and performs conversions from custom data formats;
- *biograph-etl* is related to the ETL tool you can run to populate an instance of BioGraphDB;
- *apache-httpd* contains the needed configuration’s directives to mask the Node.js and Jetty microservices behind the Apache HTTPD server;
- *apache-tinkerpop-gremlin-server-3.2.3* contains primary configurations files and some external libraries useful to enable the GraphSON [45] serialization over WebSockets;
- *biograph-fisher* is related to the three Java microservices built on-top of Jetty to compute *p*-values using the Fisher exact test.
- *biograph-node* contains the Node.js microservice which handles most of the requests to the Gremlin Server. Even if it is currently given as an all-in-one source, it is easily splittable into several little pieces, each running autonomously.
- *biograph-web* is the web application entirely written in HTML, CSS, and Javascript. All the required external libraries have been provided.

In-depth documentation will be available soon, mainly to let the users to better understand how to use Gremlin to solve bioinformatics scenarios and to extend BioGraphDB with other data sources writing new ETL modules.

## Conclusions

In this paper, we presented BioGraph, a new web application that allows to access, query, visualize and analyze biological resources belonging to different online repositories of bioinformatics and biomedical data. BioGraph building block is our previously developed graph database, called BioGraphDB, that is able to integrate and make available into a single framework heterogeneous data, including genes, proteins, miRNA, miRNA target interactions, functional annotation, pathway association and description. This way, BioGraph allows the user, using a single platform and a single query language (i.e. Gremlin), to query the BioGraphDB by means of pre-defined templates or personalized requests. In order to show the main functionalities and potentialities of the system, we presented an application scenario about functional analysis of microRNAs in breast cancer. This case study has been selected because of its biological relevance and also because it needs the use of at least four different online databases. Thanks to its modular structure, BioGraph can be easily expanded with new biological resources and updated with the latest version of the already integrated data.

## Availability and requirements

**Project name:** BioGraph

**Project homepage:**http://biograph.pa.icar.cnr.it; https://github.com/IcarPA-TBlab/BioGraph

**Operating system(s):** Unix-based, Windows, MacOS X

**Programming language:** Java, Javascript

**Other requirements:** Apache Tinkerpop, Neo4j, NodeJS, Apache HTTPD

**License:** Apache License 2.0
